# Incidence and Health Related Quality of Life of Opioid-Induced Constipation in Chronic Noncancer Pain Patients: A Prospective Multicentre Cohort Study

**DOI:** 10.1155/2018/5704627

**Published:** 2018-07-10

**Authors:** Dalila R. Veiga, Liliane Mendonça, Rute Sampaio, José C. Lopes, Luís F. Azevedo

**Affiliations:** ^1^Anesthesiology Department, Chronic Pain Center, Centro Hospitalar Universitário do Porto, Largo do Prof. Abel Salazar, 4099-001 Porto, Portugal; ^2^Centro Nacional de Observação em Dor, OBSERVDOR, Alameda Prof. Hernâni Monteiro, 4200-319 Porto, Portugal; ^3^Departamento de Biomedicina, Unidade de Biologia Experimental, Faculdade de Medicina da Universidade do Porto, Portugal; ^4^i3S, Instituto de Investigação e Inovação em Saúde, Universidade do Porto, Portugal; ^5^Instituto de Biologia Molecular e Celular (IBMC), Universidade do Porto, Portugal; ^6^Alameda Prof. Hernâni Monteiro, 4200-319 Porto, Portugal; ^7^Instituto de Biologia Molecular e Celular (IBMC), Universidade do Porto, Portugal Faculty of Medicine University of Porto, Department of Biomedicine, Porto, Portugal; ^8^Center for Health Technology and Services Research (CINTESIS); Faculdade de Medicina da Universidade do Porto, Departamento de Medicina da Comunidade Informação e Decisão em Saúde (MEDCIDS); Faculdade de Medicina da Universidade do Porto, Alameda Prof. Hernâni Monteiro, 4200-319 Porto, Portugal

## Abstract

**Background:**

High rates of opioid use for chronic noncancer pain (CNCP) have been reported worldwide, despite its association with adverse events, inappropriate use, and limited analgesic effect. Opioid-induced constipation (OIC) is the most prevalent and disabling adverse effect associated with opioid therapy. Our aim was to assess the incidence, health related quality of life (HRQOL), and disability in OIC patients.

**Methods:**

A prospective cohort study was performed, with 6 months of follow-up, of adult CNCP patients consecutively admitted in 4 multidisciplinary pain clinics (MPC). Demographic and clinical data have been collected. Brief Pain Inventory (BPI) and Short version of Treatment Outcomes in Pain Survey (S-TOPS) were used to measure functional outcomes and HRQOL. OIC was assessed using Bowel Function Index (BFI).

**Results:**

694 patients were recruited. OIC prevalence at baseline was 25.8%. At 6 months, OIC incidence was 24.8%. Female gender (OR = 1.65, *p* = 0.039), opioid therapy (OR 1.65, *p* = 0.026), and interference pain score on BPI (OR 1.10, *p* = 0.009) were identified as OIC independent predictors. OIC patients presented higher disability and pain interference and severity scores. OIC patients reported less satisfaction with outcome (*p* = 0.038).

**Discussion:**

Constipation is a common adverse event among opioid users with major functional and quality of life impairment. These findings emphasise the need of OIC adequate assessment and management.

## 1. Introduction

Chronic pain is a major public healthcare problem worldwide [1]. It has an estimated prevalence of 20% in the European population and is an important cause of quality of life impairment and substantial burden in healthcare systems [2–4]. Opioids may be a safe and reliable therapeutic option for moderate or severe chronic pain treatment, but their adequate prescription requires suitable patients' selection, clinicians' training, and patients' education. Indeed, opioid use in chronic noncancer pain (CNCP) treatment is recommended in several guidelines [5–7], and high prescription rates were observed in several countries of Europe and the United States in the last decade [8–10], although there has been a decreasing trend in prescription rates since 2011 [10, 11].

Opioid use is associated with adverse effects development such as gastrointestinal disorders, neuroendocrine dysfunction, osteoporosis, immunosuppression, cognitive disorders, drowsiness, respiratory depression, physical dependence, hyperalgesia, and addiction [12–16]. Therefore, opioid prescription requires adequate and regular clinical supervision in order to monitor their efficacy and safety.

Gastrointestinal disorders, including constipation, nausea, vomiting, abdominal cramping, and bloating, are often associated with opioid use and represent an important cause of opioid withdrawal [17]. Opioid-induced constipation, a type of secondary constipation [18], is the most prevalent and disabling adverse effect associated with chronic opioid therapy. There is no consensual definition in the literature for OIC [19]. According to Gaertner et al., OIC is defined as a “change from baseline in bowel habits and change in defecation patterns after initiating opioid therapy, which is characterized by any of the following: reduced frequency of spontaneous bowel movements (BM), development or worsening of straining to pass BMs, a sense of incomplete rectal evacuation, and harder stool consistency” [20]. One useful and reliable tool in clinical setting for OIC diagnosis is the Bowel Function Index (BFI), a self-reported patient questionnaire, with 3 variables: ease of defecation, feeling of incomplete evacuation, and personal judgment of constipation [21–23].

OIC prevalence is estimated to be as high as 40-64% in CNCP patients on opioid therapy. Moreover, OIC is the most common secondary cause of constipation to which patients do not become tolerant [24]. This prevalence may vary according to the type and dose of opioid prescribed, route of administration, and treatment duration. Morphine seems to be the opioid with the highest associated risk of OIC, while transdermal preparations of Buprenorphine and Fentanyl are associated with lower risk [25]. However, the prevalence may also vary due to differences in clinical supervision on OIC development and prophylactic laxative therapy prescription, as well as variability in patient's perception of their constipation symptoms [19, 26–28].

In clinical practice, it is important to keep a high index of suspicion concerning OIC since many patients do not feel comfortable to discuss this problem with their doctor [29].

According to the literature, the economic impact of OIC among long-term opioid users is significant [30]. Therefore, effective therapies on OIC are needed in order to reduce OIC related costs and improve patient's quality of life [30, 31]. Moreover, CNCP patients have a high prevalence of associated depression, occurring in up to 50% of these patients [32], and depression is also a prevalent condition associated with functional constipation. Because OIC has a similar clinical presentation as functional constipation, it is important for clinicians to be aware of functional constipation prevalence in this specific population when considering opioid therapy prescription. Adequate prophylactic measures and a high index of suspicion may contribute to a higher success on OIC symptoms management [17, 32, 33].

The primary aim of this study was to assess and estimate the incidence of OIC, health related quality of life and disability in CNCP patients followed in Chronic Pain Clinics. Secondary aims include assessment of pain clinical characteristics, analgesic prescription, laxative therapy, and interference in activities of daily living among OIC patients.

## 2. Methods

### 2.1. Study Design

A prospective cohort study with 6 months of follow-up was performed in 4 multidisciplinary pain clinics (MPC). This study was conducted in accordance with the Guideline for Good Clinical Practice of the International Conference on Harmonization and the ethical principles of the Declaration of Helsinki. The approvals from the National Committee for Data Protection and from local Ethical Committees were obtained. All patients were informed of the study details and signed an informed consent.

### 2.2. Patient Selection

Participants were recruited during their first appointment in one of the MPC and were included if they provided consent to participate, were 18 years or older, and presented CNCP ≥ 3 months.

Patients with psychiatric or cognitive disorders that could interfere with data collection, those physically or psychologically unable to communicate, and those unable to speak Portuguese were excluded.

### 2.3. Data Collection

Data was collected in a face to face interview with a trained interviewer at patient's first consultation in the MPC and through consultation of hospital records. Patient follow-up contacts were made by telephone by trained interviewers at 1 week and 3 and 6 months. During this period, data collection was complemented with consultation and analysis of hospital records.

A structured questionnaire was used to collect the following patients' data: demographic characteristics, clinical and pain characteristics, follow-up, pharmacological treatment prescribed, daily activities pain interference, health related quality of life (HRQOL), and clinical outcomes. The following translated, culturally adapted, and validated instruments for Portuguese population were used: Bowel Function Index (BFI) questionnaire [22, 35]; Brief Pain Inventory [BPI] [36, 37], and Short version of the Treatment Outcomes in Pain Survey (S-TOPS) [38, 39].

The BFI self-report questionnaire consists of three questions about constipation symptoms in the last 7 days: ease of defecation, feeling of incomplete evacuation, and personal judgment of constipation. Each item is scored from 0 to 100 by the patient according to the last 7 days. The BFI score is calculated as the mean of the three items scores [21, 23]. The cut-off definition BFI score for OIC is 28.8 points [34]. In cases were constipation was present at baseline, OIC was considered in patients under opioid therapy that presented a clinically significant change ≥ 12 points in BFI score, according to the criteria defined by Rentz et al. (Rentz, 2009 #2) or an increase in the dosage of previously used laxatives.

The BPI questionnaire is a self-report questionnaire measuring both the intensity of pain (sensory dimension) and interference of pain in the patient's life (reactive dimension). Each item is rated in a scale from 0 to 10. Pain severity and interference scores are calculated by the mean of the answers on each dimension of the questionnaire [35, 37, 40].

The S-TOPS questionnaire was used to assess changes in OIC related quality of life (QOL) from baseline to 6 months. S-TOPS is composed by 7 validated independent subscales with a total of 29 items: (1) Pain Symptom; (2) Physical Disability-Lower Body; (3) Physical Disability-Upper Body; (4) Family/Social Disability; (5) Role Emotional Disability; (6) Patient Satisfaction with Care; and (7) Patient Satisfaction with Outcomes. Each subscale score from S-TOPS is expressed from 0 (“no pain/disability”) to 100 (“maximum pain/disability”). The satisfaction with care and satisfaction with outcomes subscales are inverted, with 0 representing “no satisfaction” and 100 representing “maximum satisfaction”. The mean of the answers in each subscale was calculated [41].

Pain aetiology classification was conducted according to IASP Taskforce on Pain Classification for ICD-11 [42].

### 2.4. Outcome Assessment

The primary outcome assessment of our study was OIC incidence at 6 months, as previously defined, and OIC related QOL change from baseline to 6 months concerning physical disability, family and social disability, role emotional disability, and patient satisfaction with outcomes. Secondary outcomes included change from baseline in the following: pain severity and pain interference scales of the BPI, opioid consumption, laxative prescription, and general health status perception.

### 2.5. Statistical Analysis

We described our population considering OIC prevalence at baseline and pain clinical characterization. The primary analysis was stratified on OIC incidence at 6 months of follow-up. Descriptive statistics of patient characteristics and clinical variables are presented as frequencies with percentages (%), median with interquartile range or mean with standard deviation [43] when appropriate. Nonparametric and parametric tests were performed for comparisons between numerical variables according their distribution and chi-squared test for categorical variables.

A *p*-value ≤ 0.05 was considered significant for all tests.

Multivariate logistic regression analysis was performed. Univariate predictors of outcome with a *p*-value <0.10 were selected for multivariate logistic regression analysis with stepwise backward elimination. The dependent variable was OIC and possible predictors were used as independent variables. We have evaluated goodness of fit of the logistic regression model using the Hosmer-Lemeshow test and receiver operating characteristic (ROC) curve to evaluate its predictive and discriminative power.

Statistical analysis was performed using the Statistical Package for the Social Sciences version 24.0 (SPSS, USA).

## 3. Results

### 3.1. Prevalence and Incidence of OIC

694 patients with CNCP diagnosis were recruited. The general characteristics of the cohort are described in Table 1.

OIC prevalence at baseline was 25.8% (*n* = 174; BFI median 18.9 ± 30.9). At their first consultation, OIC patients were mostly >75 years old (*n* = 29, 33.0%), female (*n* = 131, 75.3%), with a low level of education (1-4 years; *n* = 94, 54.3%), and with a median BMI of 26.3 ± 3.8. 63.2% of OIC patients (*n* = 110) were prescribed with weak opioids and 36.8% (*n* = 54) with strong opioids. Most OIC patients were not prescribed with laxative therapy (*n* = 162; 93.1%) before their first consultation at MPC. At six-month follow-up, OIC was persistent in 51.7% (*n* = 90) of patients having OIC at baseline and resolved in 48.3% (*n* = 84) of them. There were no significant differences among these two subgroups except concerning prescription of laxative therapy in persistent OIC patients (*p* < 0.001) (Table 2).

At 6 months, OIC prevalence was 32.0% (*n* = 216), and OIC incidence was 24.8% (*n* = 126; BFI mean 18.8 ± 28.5). OIC patients at 6 months were mostly 40-65 years old (*n* = 60, 35.9%), female (*n* = 134, 80.2%), with low levels of education (1-4 years; *n* = 94, 56.3%), and with a median BMI of 26.9 ± 5.1. There was a significant association among OIC development and opioid prescription (*p* = 0.034), but there were no significant differences among strong and weak opioids prescriptions in these patients. Patients with OIC had a higher prescription rate of laxative therapy (*p* < 0.001).

Although other adverse drug reactions [44] associated with opioids were not our primary focus, we obtained data regarding other ADR, at 6 months, for 145 patients. Among those, the most frequently reported ADRs associated with opioids, besides OIC, were nausea and vomiting (27%), rash (11%), somnolence/sleepiness (10%), dizziness (10%), and pruritus (9%).

### 3.2. Clinical Characteristics, Severity, and Interference of Pain in OIC Patients

Pain characteristics are described in Table 4.

**Figure 1:**
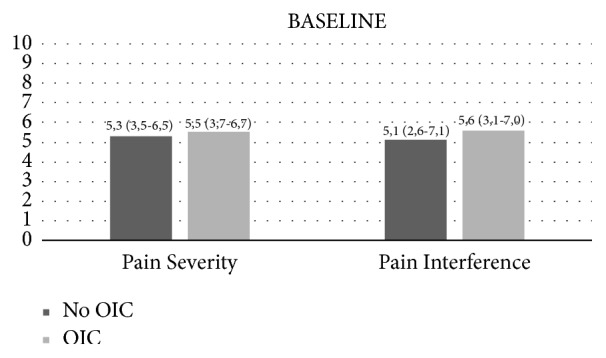
Brief Pain Inventory questionnaire at baseline. Data are presented as median (interquartile range). *P* values are derived from T test (pain severity) and Mann-Whitney U test (pain interference),

Compared to non-OIC patients, OIC patients had longer pain duration and presented more often pain at cervical region, lumbar region, upper limbs, and lower limbs, both at baseline and at 6 months of follow-up. More frequent pain aetiologies were chronic musculoskeletal pain and chronic neuropathic pain. However, these were not statistically different from non-OIC patients except for pain location at cervical region (baseline and 6 months) and upper limb (baseline). There were no significant differences in pain severity and interference scores at baseline among OIC and non-OIC patients (Figure 1). However, at baseline OIC patients reported statistically significant higher interference pain scores in general activity, mood, walking ability, normal work, relations with other people, sleep, and enjoyment of life (*p* < 0.05).

**Figure 2:**
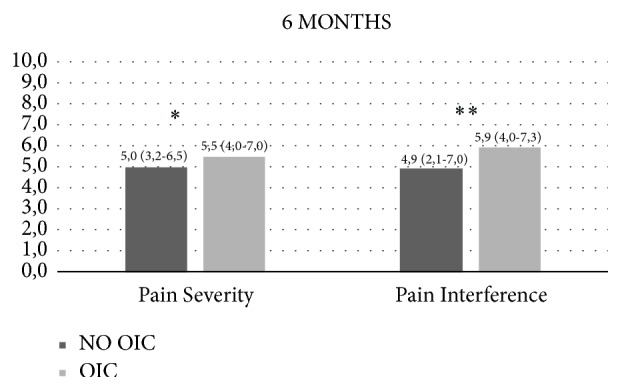
Brief Pain Inventory at 6 months. Data are presented as median (interquartile range). *P* values are derived from T test (Pain severity) and Mann-Whitney U test (Pain interference). ^*∗*^*p* < 0.05, ^*∗∗*^*p* < 0.001.

At 6-month follow-up, OIC patients presented significant higher pain severity scores (5.5 P25-P75 4.0-7.0 versus 5.0 P25-P75 3.2-6.5, *p* = 0.021) and higher pain interference scores (5.9 P25-P75 4.0-7.3 versus 4.9 P25-P75 2.1-7.0, *p* < 0.001) in all domains (Figure 2).

### 3.3. Outcomes Assessment of OIC and Related Quality of Life (QoL)

**Figure 3:**
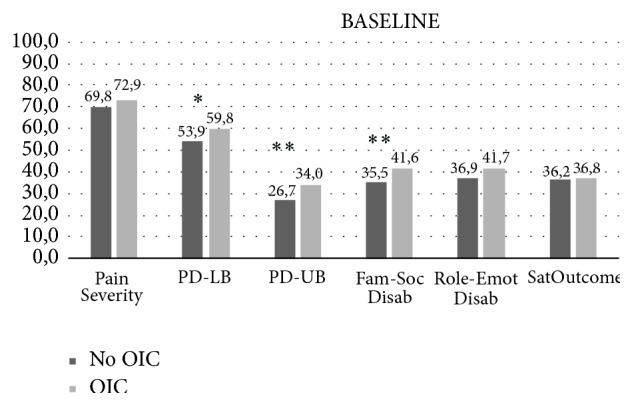
Short-Treatment Outcomes in Pain Survey (S-TOPS) questionnaire at baseline. OIC: opioid-induced constipation; PD-LB: physical Disability lower body; PD-LB: physical disability upper body; FAM-SOC DISAB: family and social disability; ROLE-EMOT DISAB: role emotional disability; SATOUTCOME: satisfaction with outcomes. Data are presented as median of each subscale. *P* values are derived from Mann-Whitney U test comparisons. ^*∗*^*p* < 0.05, ^*∗∗*^*p* < 0,001.

At baseline, OIC patients reported significantly higher disability in physical lower function (59.8 ± 30.9 versus 53.9 ± 32.5, *p* = 0.028), physical upper function (34.0 ± 24.0 versus 26.7 ± 21.5, *p* < 0.001), and family/social disability (41.6 ± 24.9 versus 35.5 ± 24.7, *p* < 0.001) (Figure 3), and 32.9% (*n* = 222) considered that their health was poor.

**Figure 4:**
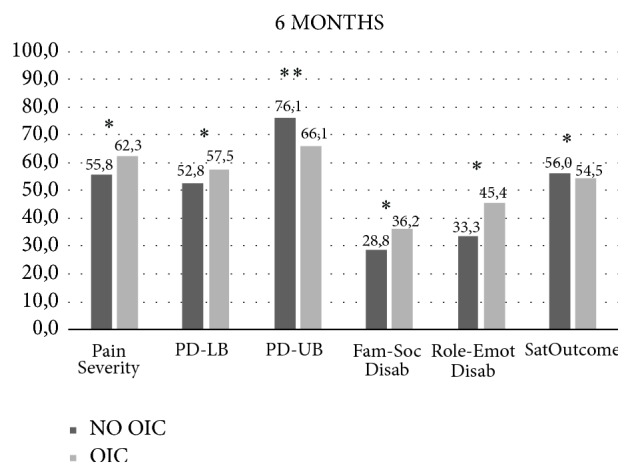
Short-Treatment Outcomes in Pain Survey (S-TOPS) questionnaire at 6 months. OIC: opioid-induced constipation; PD-LB: physical disability lower body; PD-LB: physical disability upper body; FAM-SOC DISAB: family social disability; ROLE-EMOT DISAB: role emotional disability; SATOUTCOME: satisfaction with outcomes. Data are presented as median of each subscale. *P* values are derived from Mann-Whitney U test comparisons. ^*∗*^*p* < 0.05, ^*∗∗*^*p* < 0.001.

At 6 months, OIC patients reported significantly higher disability in all subscales of S-TOPS questionnaire except physical upper function: pain severity (62.3 ± 20.9 versus 55.8 ± 25.2, *p* = 0.011), physical lower function (57.5 ± 31.1 versus 52.8 ± 33.7, *p* = 0.026), family/social disability (36.2 ± 27.5 versus 28.8 ± 26.8, *p* = 0.005), and role emotional disability (45.4 ± 34.5 versus 53.9 ± 32.5, *p* = 0.028). OIC patients reported significantly less satisfaction with outcomes than non-OIC patients (54.5 ± 22.0 versus 56.0 ± 26.1, *p* = 0.038) (Figure 4). At 6 months of follow-up, 38.0% (*n* = 256) of OIC patients considered that their health was poor.

### 3.4. Predictors of OIC at 6 Months

Tables 3 and 4 and Figure 2 present factors associated with OIC incidence development at 6 months. Gender (*p* = 0.009), opioid use (*p* = 0.034), BPI severity score (*p* = 0.021), and BPI interference score (*p* < 0.001) are significantly associated with OIC. Taking into account the available literature [28, 40, 41], we decided to include depressive disorders diagnosis in our multivariate regression analysis model.

Table 5 describes independent predictors of OIC at 6 months identified in a multivariate logistic regression analysis with stepwise backward elimination. In our multivariate model, opioid therapy (OR 1.65, *p* = 0.026), female gender (OR 1.65, *p* = 0.039), and higher interference pain score (OR 1.10, *p* = 0.009) are independent predictors of higher risk of OIC at 6 months. Our model had a good predictive power (Hosmer and Lemeshow test: *p* = 0.107) and a moderate discriminative power (AUC 0,678, *p* < 0.001).

## 4. Discussion

Opioid-induced constipation is a prevalent and debilitating condition with a significantly high impact in patient's quality of life and well-being. OIC has an estimated prevalence of 15-90 % in chronic noncancer pain patients. Our estimated prevalence of 25.8% at baseline and 32.0% at 6-month follow-up is in accordance with previous data [19, 27]. Indeed, in the last decades there has been an increment of opioid prescription for chronic noncancer pain management that may explain high OIC prevalence among this specific population [6, 15, 45]. Long-term exposure, high opioid doses and absence of laxative therapy are some of the conditions associated with OIC development [17, 27].

Chronic noncancer pain is more prevalent in female gender and with increasing age [6]. Therefore, a similar demographic data in the OIC population was expected [46, 47].

In our study, at 6 months OIC patients reported higher pain severity score (*p* = 0.021) and higher pain interference score (*p* < 0.001). These results are in accordance with the statistically significant association with opioid prescription in these patients (*p* = 0.034). Indeed, it is expected that patients with more severe pain are more often prescribed with opioids and for longer periods, and, therefore, present a higher risk of OIC development. Several studies have reported the association of OIC with the burden of opioid prescription in CNCP in the last decades [1, 48, 49].

OIC is the most prevalent adverse effect associated with opioid use and has severe impact on patient's quality of life and ability to perform daily life activities and work [27, 50]. OIC results from opioid action on mu-opioid receptors in the gastrointestinal tract that induces delayed gastric emptying, decreased bowel movements, increased intestinal fluids absorption, and increased anal sphincter tone [17]. However, opioids may present different effects depending on their location in the gastrointestinal tract. For instance, there is tolerance development for mu-opioid receptor actions for all gastrointestinal tract except for colon [51]. Therefore, while other gastrointestinal disorders tend to disappear in long-term opioid exposure, constipation is a persistent and debilitating side effect that must be appropriately treated [51, 52]. Moreover, OIC is such a distressing adverse event that, as a consequence, some patients prefer to reduce or discontinue their opioid therapy and be in pain rather than experiencing severe constipation.

OIC symptoms management is challenging and often with unsatisfactory response to the available therapeutics. OIC treatment encompasses either nonpharmacological approaches (dietary fibre, fluid intake, and physical activity) or nonspecific pharmacological approaches with laxatives use (stimulants, softeners, bulk forming, and enemas) and prokinetics. However, even with these therapeutic measures many patients do not have significant OIC symptoms relief. LoCasale et al. reported in a prospective longitudinal study of 489 OIC noncancer pain patients, a high rate (48%) of inadequate OIC relief despite adequate laxative therapy prescribed [24]. Nowadays, new specific pharmacological treatments with formulations containing peripherally acting opioid antagonists such as methylnaltrexone bromide and oxycodone/naloxone are available and may allow more efficacy in OIC management without analgesia impairment [26, 27, 53, 54]. However, in spite of the available recommendations, early prescription of prophylactic measures such as dietary fibre, fluid intake, physical activity, and laxative use are not routine when starting an opioid prescription [24, 27, 53]. In our study, the prescription rate of laxative therapy at baseline was only of 6.9% (*n* = 12) in spite of a prevalence of opioid use of 59.6% (*n* = 402) and an OIC prevalence of 25.8% (*n* = 174). At 6 months of follow-up, our prescription rate of laxative therapy in patients with persistent OIC was still low (*n* = 19, 16.7%). These results may reflect the lack of awareness of healthcare professionals about the real impact of OIC on patient's quality of life and well-being. More efforts should be made to promote physician's education on OIC and increase safe and responsible opioid use.

At six-month follow-up, OIC patients reported significantly higher disability in all subscales of S-TOPS questionnaire except physical upper function, which is consistent with previous studies [20, 27]. Indeed, OIC has a severe and debilitating repercussion in the patients' quality of life and impairs their ability to perform daily life activities. In our sample, OIC patients reported significantly less satisfaction with outcomes than non-OIC patients (*p* = 0.034). LoCasale et al. reported in another prospective observational study of six-month follow-up that OIC patients despite adequate laxative therapy prescription presented persistent constipation and a small improvement in their quality of life and ability to perform daily life activities [55]. This may lead patients to reduce their opioid analgesic doses or even to opioid withdrawal, which can compromise their CNCP management. Another additional problem is related to patients' long-term exposure to opioid therapy and, therefore, to persistent OIC symptoms and associated morbidity with significant economic and societal costs [30, 31, 50, 56].

We have identified as independent predictors of incident OIC at six-month follow-up, female gender, higher interference pain score on BPI, and opioid therapy. The latter finding was expected since opioid therapy is the major risk factor for constipation development in CNCP patients. Moreover, this risk increases with higher doses and longer exposure times and varies with the type of opioid prescribed and route of administration [19, 25, 27]. On the other hand, patients with higher interference pain scores are those at increased risk of being prescribed with opioids and in higher doses in order to control their pain. Therefore, it was also expected that patients with higher severity pain scores were also associated with higher risk of OIC development at 6 months. However, we have not found this association, which may be explained by a more significant difference of interference than severity pain scores among OIC and non-OIC patients. Taking into account the higher prevalence of CNCP in female gender and with increasing age, it is reasonable to expect that OIC also presents a similar demographic characterization [25, 46]. To the best of our knowledge, there has been only one publication concerning OIC predictors' identification in a sample of 2324 Italian patients on chronic pain treatment [57]. According to the results of this study, opioid use, female gender, and increasing age are risk factors for OIC development. In cancer pain patients the risk of OIC development seems to be higher when higher opioid doses are needed.

Our study has several strengths. It was an observational prospective study with 6-month follow-up period regarding OIC incidence, its predictive factors, functional outcomes, and quality of life assessment based in the seven subscales of the S-TOPS questionnaire following IMMPACT recommendations [58]. We provided a detailed assessment based on standardized and validated questionnaires. We believe that our cohort is representative of the CNCP population taking into account the fact that we included data from 4 different MPC. This study also presents some limitations. As a prospective observational study, it may present selection bias from losses of follow-up. To overcome this, we have developed retention strategies since eligible patients have consented and those who have continued for all duration of the study in order to reduce our follow-up losses.

## 5. Conclusions

Opioid use is associated with the potential risk of adverse effects development. OIC is the most prevalent side effect and may negatively impact patients' adherence to their analgesic prescription. It is important to promote clinician's awareness on OIC since it is still a grossly underrecognized and undertreated condition. A high index of suspicion in high risk populations such as CNCP patients, utilization of adequate diagnostic tools, and routine implementation of prophylactic therapies such as laxatives prescription are key measures to improve OIC management and reduce its associated disability and costs.

## Figures and Tables

**Table 1:** General characteristics of CNCP patients at baseline.

**Variable**		**No Constipation**	**Constipation**
**Total**	674 (100%)	500 (74.2%)	174 (25.8%)

**Age, years**			
18-45	151	110 (22.0%)	41 (23.6%)
45-60	214	162 (32.4%)	52 (29.9%)
60-75	221	169 (33.8%)	52 (29.9%)
>75	88	59 (11.8%)	29 (16.7%)

**Gender**			
Female	488	357 (71.4%)	131 (75.3%)
Male	186	143 (28.6%)	43 (24.7%)

**BMI**	27.0 (±5.20)	27.1 (±5.20)	26.5 (±5.20)

**Education level**			
No education	19	12 (2.4%)	7 (4.0%)
1-4 years (basic 1st cycle)	340	246 (49.2%)	94 (54.3%)
5-9 years (basic 2nd and 3rd cycles)	163	127 (25.4%)	36 (20.8%)
10-12 years (secondary)	78	60 (12.0%)	18 (10.4%)
More than 12 years(higher)	73	55 (11.0%)	18 (10.4%)

**Opioid therapy**	402	228 (56.7%)	174 (43.2%)

**Analgesic prescription**			
Non-opioid	213	163 (32.6%)	50 (28.7%)
Weak opioid	339	229 (45.8%)	110 (63.2%)
Strong opioid	126	62 (12.4%)	64 (36.8%)

**Laxatives use**	24	12 (15.8%)	12 (30.8%)

**Current depressive disorder on treatment**	139	97 (20.8%)	42 (25.8%)

OIC: opioid-induced constipation; BMI: body mass index; data are presented as *n* (%) except BMI which is presented as mean ± standard deviation. Proportions are calculated as column proportions.

**Table 2:** Follow-up at 6 months of patients with OIC at baseline.

	**6 Months Follow-up of OIC patients at baseline**		
**Variable**	Previous OIC (OIC absent at 6 months)	Current OIC (OIC still present at 6 months)	*P* value

**Total**	84 (48.3%)	90 (51.7%)	

**Age, years**			0.349^a^
18-45	23 (27.4%)	18 (20.0%)	
45-60	27 (32.1%)	25 (27.8%)	
60-75	20 (16.7%)	32 (35.6%)	
>75	14 (48.3%)	15 (16.7%)	

**Gender**			
Female	60 (71.4%)	71 (78.9%)	0.293^a^
Male	24 (28.6%)	19 (21.1%)	

**BMI **	27.15	26.49	0.178^b^
Mean [34];	(±5.20)	(±5.20)	

**Education level**			** 0.047** ^a^
No education	4 (4.8%)	3 (3.3%)	
1-4 years (basic 1st cycle)	40 (48.2%)	54 (60.0%)	
5-9 years (basic 2nd and 3rd cycles)	15 (18.1%)	21 (23.3%)	
10-12 years (secondary)	12 (14.5%)	6 (6.7%)	
More than 12 years(higher)	12 (14.5%)	6 (6.7%)	

**Opioid therapy**			0.409^a^
Weak opioid	43 (51.2%)	50 (55.6%)	
Strong opioid	41 (48.8%)	40 (44.4%)	

**Laxatives use**	0 (0%)	19 (100%)	**<0.001** ^a^

**Current depressive disorder on treatment**	20 (24.4%)	14 (27.2%)	0.686

OIC: opioid-induced constipation; BMI: body mass index. Data are presented as *n* (%) except BMI which is presented as mean ± standard deviation. Proportions are calculated as column proportions. *P* values are derived from (a) x2 comparisons; (b) *T* test.

**Table 3:** OIC incidence at 6 months.

	**OIC Incidence at 6 months of follow-up**		
**Variable**	**No Constipation**	**Constipation**	*P* value

**Age, years**			0.171^a^
18-45	123 (24.3%)	28 (16.8%)	
45-60	154 (30.4%)	60 (35.9%)	
60-75	167 (32.9%)	54 (32.3%)	
>75	63 (12.4%)	25 (15.0%)	

**Gender**			
Female	354 (69.8%)	134 (80.2%)	**0.009** ^a^
Male	153 (30.2%)	33 (19.8%)	

**BMI**	27.33 (5.50)	26.87 (5.10)	0.357^b^

**Education level **			0.562^a^
No education	15 (3.0%)	4 (2.4%)	
1-4 years (basic 1st cycle)	246 (48.6%)	94 (56.3%)	
5-9 years (basic 2nd and 3rd cycles)	127 (25.1%)	36 (21.6%)	
10-12 years (secondary)	61 (12.1%)	17 (10.2%)	
More than 12 years (higher)	57 (11.3%)	16 (9.6%)	

**Opioid therapy**			**0.034** ^a^
Yes	338 (72.8%)	126 (75.4%)	
No	169 (80.5%)	41 (24.6%)	

**Analgesic Therapy**			0.087^a^
Non-opioid	168 (80.4%)	41 (24.6%)	
Weak opioid	233 (71.9%)	91 (54.5%)	
Strong opioid	104 (74.8%)	35 (21.0%)	

**Laxatives use**	2 (100%)	51 (100%)	**<0.001** ^a^

**Depressive disorder **	102 (21.4%)	37 (24.0%)	0.504^a^

OIC: opioid-induced constipation; BMI: body mass index. Data are presented as *n* (%) except BMI which is presented as mean ± standard deviation. Proportions are calculated as column proportions. *P* values are derived from (a) x2; (b) *T* test comparisons.

**Table 4:** Pain characteristics.

**Pain Characteristics**			**Baseline**			**6 Months**		
		**Total**	**No constipation**	**Constipation**	**P value**	**No Constipation**	**Constipation**	**P value**
**Pain duration**	Pain duration in years	4.0 (2.0-12.0)	4.0 (2.0-13.0)	5.0 (2.0-10.5)	0.207^c^	4.0 (2.0-12.0)	4.7 (2.0-14.0)	0.782^b^

**Pain location**	Head	67 (65.0%)	67 (65.0%)	36 (35.0%)	**0.021** ^a^	69 (67.0%)	34 (33.0%)	0.931^a^
	Face	26 (61.9%)	26 (61.9%)	16 (38.1%)	0.060^a^	28 (66.7%)	14 (33.3%)	0.921^a^
	Cervical region	172 (68.5%)	172 (68.5%)	79 (31.5%)	**0.010** ^a^	156 (62.2%)	95 (37.8%)	**0.028** ^a^
	Dorsal region	41 (64.1%)	41 (64.1%)	23 (35.9%)	0.052^a^	46 (71.9%)	18 (28.1%)	0.485^a^
	Lumbar region	292 (73.7%)	292 (73.7%)	104 (26.3%)	0.324^a^	265 (66.9%)	131 (33.1%)	0.803^a^
	Abdominal region	21 (73.8%)	21 (73.8%)	11 (26.2%)	0.954^a^	28 (66.7%)	14 (33.3%)	0.921^a^
	Upper Limb	240 (70.4%)	240 (70.4%)	101 (29.6%)	**0.022** ^a^	226 (66.3%)	115 (31.5%)	0.566^a^
	Lower Limb	314 (72.5%)	314 (72.5%)	119 (27.5%)	0.185^a^	294 (67.9%)	139 (33.6%)	0.732^a^

**Pain aetiology**	Chronic primary pain	50 (7.4%)	32 (64.0%)	18 (36.0%)	0.087^a^	18 (43.9%)	23 (56.1%)	**0.008** ^a^
	Chronic postsurgical and posttraumatic pain	92 (13.6%)	66 (71,7%)	26 (28.3%)	0.564^a^	47 (62.7%)	28 (37.3%)	0.813^a^
	Chronic neuropathic pain	170 (25.2%)	128 (75.3%)	42 (24.7%)	0.702^a^	99 (67.8%)	47 (32.2%)	0.926^a^
	Chronic headache and orofacial pain	15 (2.2%)	6 (40.0%)	9 (60.0%)	**0.002** ^a^	7 (58.3%)	5 (41.7%)	0.581^a^
	Chronic visceral pain	26 (3.9%)	15 (57.7%)	11 (42.3%)	0.050^a^	14 (63.6%)	8 (36.4%)	0.833^a^
	Chronic musculoskeletal pain	254 (37.7%)	183 (72%)	71 (28.0%)	0.324^a^	249 (69.9%)	107 (30.1%)	0.310^a^

Data are presented as *n* (%) except pain duration which is presented as median (interquartile range). Proportions are calculated as row proportions. *P* values are derived from (a) x^2^ comparisons; (b) *T* test; (c) Mann-Whitney *U* test.

**Table 5:** Predictors of OIC at 6 months.

	**Crude OR (95%CI)**	**Adjusted** **OR** **(95%CI)**
**Gender**	**p** = 0.010	**p** = 0.039
Female	1.76 (1.15-2.69)	1.65 (1.03-2.67)
Male	1	1

**Current depressive disorders**	*P* = 0.50	-
	1.16 (0.76-1.78)	

**Opioid therapy**	**p** = 0.036	**p** = 0.026
Yes	1.53 (1.03-2.28)	1.65 (1.06-2.56)
No	1	1

**Severity Scale**	*P* = 0.058	-
	1.06 (1.00-1.13)	

**Interference Scale**	**p** = 0.001	**p** = 0.009
	1.13 (1.06-1.21)	1.10 (1.02-1.19)

95% CI, 95% confidence interval; OR, odds ratio. Predictors of opioid induced constipation defined by simple and multiple logistic regression; crude and adjusted ORs for categories of demographic variables, opioid therapy, and pain characteristics variables. Adjusted ORs were calculated using multivariate logistic regression models. Multivariate model included adjustment all variables with crude association measures with *P* values < 0.1 in the univariate analysis. *P* values for the omnibus tests evaluating the significance of each predictor variable.

## Data Availability

The data used to support the findings of this study are available from the corresponding author upon request.

## Abbreviations

BFI: Bowel Function Index
BPI: Brief Pain Inventory
CNCP: Chronic noncancer pain
CI: Confidence interval
IASP: International Association for the Study of Pain
ICD: International Classification of Diseases
IMMPACT: Initiative on Methods, Measurement, and Pain Assessment in Clinical Trials
MCPC: Multidisciplinary Chronic Pain Clinics
OIC: Opioid-induced constipation
P25: 25th percentile
P75: 75th percentile
SD: Standard deviation
S-TOPS: Shortened Treatment Outcomes in Pain Survey.

## References

[1] Goldberg D. S., McGee S. J. (2011). Pain as a global public health priority. *BMC Public Health*.

[2] Breivik H., Eisenberg E., O'Brien T. (2013). The individual and societal burden of chronic pain in Europe: the case for strategic prioritisation and action to improve knowledge and availability of appropriate care. *BMC Public Health*.

[3] Azevedo L. F., Costa-Pereira A., Mendonça L., Dias C. C., Castro-Lopes J. M. (2016). The economic impact of chronic pain: a nationwide population-based cost-of-illness study in Portugal. *European Journal of Health Economics*.

[4] Azevedo L. F., Costa-Pereira A., Mendonça L., Dias C. C., Castro-Lopes J. M. (2013). Chronic pain and health services utilization: Is there overuse of diagnostic tests and inequalities in nonpharmacologic treatment methods utilization?. *Medical Care*.

[5] Chapman C. R., Lipschitz D. L., Angst M. S. (2010). Opioid pharmacotherapy for chronic non-cancer pain in the United States: A research guideline for developing an evidence-base. *The Journal of Pain*.

[6] O'Brien T., Christrup L. L., Drewes A. M. (2017). European Pain Federation position paper on appropriate opioid use in chronic pain management. *European Journal of Pain*.

[7] Chou R., Fanciullo G. J., Fine P. G. (2009). Clinical guidelines for the use of chronic opioid therapy in chronic noncancer pain. *The Journal of Pain*.

[8] CDC guidelines for Prescribing Opioids for Chronic Pain— United States, 2016

[9] van Amsterdam J., van den Brink W. (2015). The misuse of prescription opioids: A threat for Europe?. *Current Drug Abuse Reviews*.

[10] Häuser W., Schug S., Furlan A. D. (2017). The opioid epidemic and national guidelines for opioid therapy for chronic noncancer pain. *PAIN Reports*.

[11] Pezalla E. J., Rosen D., Erensen J. G., Haddox J. D., Mayne T. J. (2017). Secular trends in opioid prescribing in the USA. *Journal of Pain Research*.

[12] Noble M., Tregear S. J., Treadwell J. R., Schoelles K. (2008). Long-Term Opioid Therapy for Chronic Noncancer Pain: A Systematic Review and Meta-Analysis of Efficacy and Safety. *Journal of Pain and Symptom Management*.

[13] Raghavan S., Harvey A. D., Humble S. R. (2011). New opioid side effects and implications for long-term therapy. *Trends in Anaesthesia and Critical Care*.

[14] Duarte R., Raphael J. (2014). The pros and cons of long-term Opioid therapy. *Journal of Pain and Palliative Care Pharmacotherapy*.

[15] Blake H., Leighton P., van der Walt G., Ravenscroft A. (2015). Prescribing opioid analgesics for chronic non-malignant pain in general practice—a survey of attitudes and practice. *British Journal of Pain*.

[16] Ballantyne J. C. (2015). Opioid Therapy in Chronic Pain. *Physical Medicine and Rehabilitation Clinics of North America*.

[17] Nelson A. D., Camilleri M. (2016). Opioid-induced constipation: advances and clinical guidance. *Therapeutic Advances in Chronic Disease*.

[18] Hsieh C. (2005). Treatment of constipation in older adults. *American Family Physician*.

[19] Panchal S. J., Müller-Schwefe P., Wurzelmann J. I. (2007). Opioid-induced bowel dysfunction: prevalence, pathophysiology and burden. *International Journal of Clinical Practice*.

[20] Gaertner J., Siemens W., Camilleri M. (2015). Definitions and outcome measures of clinical trials regarding opioid-induced constipation: A systematic review. *Journal of Clinical Gastroenterology*.

[21] Abramowitz L., Béziaud N., Caussé C., Chuberre B., Allaert F. A., Perrot S. (2013). Further validation of the psychometric properties of the Bowel Function Index for evaluating opioid-induced constipation (OIC). *Journal of Medical Economics*.

[22] Dueñas M., Mendonça L., Sampaio R. (2017). Reliability and validity of the Bowel Function Index for evaluating opioid-induced constipation: translation, cultural adaptation and validation of the Portuguese version (BFI-P). *Current Medical Research and Opinion*.

[23] Ueberall M. A., Müller-Lissner S., Buschmann-Kramm C., Bosse B. (2011). The bowel function index for evaluating constipation in pain patients: Definition of a reference range for a non- constipated population of pain patients. *Journal of International Medical Research*.

[24] LoCasale R. J., Datto C., Margolis M. K., Coyne K. S. (2016). Satisfaction with Therapy Among Patients with Chronic Noncancer Pain with Opioid-Induced Constipation. *Journal of managed care & specialty pharmacy*.

[25] Müller-Lissner S., Bassotti G., Coffin B. (2017). Opioid-induced constipation and bowel dysfunction: A clinical guideline. *Pain Medicine*.

[26] Siemens W., Gaertner J., Becker G. (2015). Advances in pharmacotherapy for opioid-induced constipation - A systematic review. *Expert Opinion on Pharmacotherapy*.

[27] Kumar L., Barker C., Emmanuel A. (2014). Opioid-Induced Constipation: Pathophysiology, Clinical Consequences, and Management. *Gastroenterology Research and Practice*.

[28] Chang H. Y., Lembo A. J. (2008). Opioid-induced bowel dysfunction. *Current Treatment Options in Gastroenterology*.

[29] LoCasale R. J., Datto C., Wilson H., Yeomans K., Coyne K. S. (2016). The Burden of Opioid-Induced Constipation: Discordance Between Patient and Health Care Provider Reports. *Journal of managed care & specialty pharmacy*.

[30] Alemayehu B., Coyne K., King F. (2014). Self Reported Health Care Resource Use and Indirect Economic Burden of Opioid Induced Constipation (Oic). *Value in Health*.

[31] Wan Y., Corman S., Gao X., Liu S., Patel H., Mody R. (2015). Economic burden of opioid-induced constipation among long-term Opioid users with non cancer pain. *American Health and Drug Benefits*.

[32] Barkin R. L., Barkin S. J., Irving G. A., Gordon A. (2011). Management of chronic noncancer pain in depressed patients. *Postgraduate Medical Journal*.

[33] Hosseinzadeh S. T., Poorsaadati S., Radkani B., Forootan M. (2011). Psychological disorders in patients with chronic constipation. *Gastroenterology and Hepatology from Bed to Bench*.

[34] Ahmedzai S. H., Nauck F., Bar-Sela G., Bosse B., Leyendecker P., Hopp M. (2012). A randomized, double-blind, active-controlled, double-dummy, parallel-group study to determine the safety and efficacy of oxycodone/naloxone prolonged-release tablets in patients with moderate/severe, chronic cancer pain. *Palliative Medicine*.

[35] Azevedo L. F., Pereira A. C., Dias C., Romão J. (2007). Translation, cultural adaptation and multicentric validation study of chronic pain screening and impact assessment instruments. *DOR*.

[36] Furler L. (2013). Validity and reliability of the pain questionnaire "Brief Pain Inventory". A literature research. *Pflegezeitschrift*.

[37] Keller S., Bann C. M., Dodd S. L., Schein J., Mendoza T. R., Cleeland C. S. (2004). Validity of the brief pain inventory for use in documenting the outcomes of patients with noncancer pain. *The Clinical Journal of Pain*.

[38] Dworkin R. H., Turk D. C., Farrar J. T. (2005). Core outcome measures for chronic pain clinical trials: IMMPACT recommendations. *PAIN*.

[39] Mulla S. M., Maqbool A., Sivananthan L. (2015). Reporting of IMMPACT-recommended core outcome domains among trials assessing opioids for chronic non-cancer pain. *PAIN*.

[40] Tan G., Jensen M. P., Thornby J. I., Shanti B. F. (2004). Validation of the brief pain inventory for chronic nonmalignant pain. *The Journal of Pain*.

[41] Haroutiunian S., Donaldson G., Yu J., Lipman A. G. (2012). Development and validation of shortened, restructured Treatment Outcomes in Pain Survey instrument (the S-TOPS) for assessment of individual pain patients' health-related quality of life. *PAIN*.

[42] Treede R.-D., Rief W., Barke A. (2015). A classification of chronic pain for ICD-11. *PAIN*.

[43] Ortiz-Catalan M., Guðmundsdóttir R. A., Kristoffersen M. B. (2016). Phantom motor execution facilitated by machine learning and augmented reality as treatment for phantom limb pain: a single group, clinical trial in patients with chronic intractable phantom limb pain. *The Lancet*.

[44] Headrick J. P., Pepe S., Peart J. N. (2012). Non-analgesic effects of opioids: Cardiovascular effects of opioids and their receptor systems. *Current Pharmaceutical Design*.

[45] Ballantyne J. C. (2010). Opioid controls: Regulate to educate. *Pain Medicine*.

[46] Campbell C. I., Weisner C., LeResche L. (2010). Age and gender trends in long-term opioid analgesic use for noncancer pain. *American Journal of Public Health*.

[47] Almakadma Y. S., Simpson K. (2013). Opioid therapy in non-cancer chronic pain patients: Trends and efficacy in different types of pain, patients age and gender. *Saudi Journal of Anaesthesia*.

[48] Boudreau D., Von Korff M., Rutter C. M. (2009). Trends in long-term opioid therapy for chronic non-cancer pain. *Pharmacoepidemiology and Drug Safety*.

[49] Foy R., Leaman B., McCrorie C. (2016). Prescribed opioids in primary care: Cross-sectional and longitudinal analyses of influence of patient and practice characteristics. *BMJ Open*.

[50] Coyne K. S., Margolis M. K., Yeomans K. (2015). Opioid-Induced Constipation Among Patients with Chronic Noncancer Pain in the United States, Canada, Germany, and the United Kingdom: Laxative Use, Response, and Symptom Burden Over Time. *Pain Medicine*.

[51] Akbarali H. I., Inkisar A., Dewey W. L. (2014). Site and mechanism of morphine tolerance in the gastrointestinal tract. *Neurogastroenterology & Motility*.

[52] Pasternak G. W. (2005). Molecular biology of opioid analgesia. *Journal of Pain and Symptom Management*.

[53] Holzer P. (2008). New approaches to the treatment of opioid-induced constipation. *European Review for Medical and Pharmacological Sciences*.

[54] Reichle F. M., Conzen P. F. (2008). Methylnaltrexone, a new peripheral *μ*-receptor antagonist for the prevention and treatment of opioid-induced extracerebral side effects. *Current Opinion in Infectious Diseases*.

[55] Locasale R. J., Datto C. J., Margolis M. K., Tack J., Coyne K. S. (2015). The impact of opioid-induced constipation among chronic pain patients with sufficient laxative use. *International Journal of Clinical Practice*.

[56] Holliday S. M., Hayes C., Dunlop A. J. (2017). Does brief chronic pain management education change opioid prescribing rates? A pragmatic trial in Australian early-career general practitioners. *PAIN*.

[57] Rosti G., Gatti A., Costantini A., Sabato A. F., Zucco F. (2010). Opioid-related bowel dysfunction: Prevalence and identification of predictive factors in a large sample of Italian patients on chronic treatment. *European Review for Medical and Pharmacological Sciences*.

[58] Dworkin R. H., Turk D. C., Peirce-Sandner S. (2012). Considerations for improving assay sensitivity in chronic pain clinical trials: IMMPACT recommendations. *PAIN*.

